# Teaching evidence based practice in physical therapy in a developing country: a national survey of Philippine schools

**DOI:** 10.1186/1472-6920-13-154

**Published:** 2013-11-22

**Authors:** Edward James R Gorgon, Mark David S Basco, Almira T Manuel

**Affiliations:** 1 Department of Physical Therapy, College of Allied Medical Professions, University of the Philippines Manila, Pedro Gil Street, Malate 1004, Manila, Philippines; 2 Physical Therapy Department, Muenster Memorial Hospital, 605 North Maple Street, Muenster, TX, USA; 3 Department of Physical Therapy, Emilio Aguinaldo College, San Marcelino Street, Malate 1000, Manila, Philippines

**Keywords:** Allied health, Evidence based health care, Medical education, Teaching methods, Under-developed countries

## Abstract

**Background:**

Early education on the foundations of evidence based practice (EBP) is advocated as a potent intervention toward enhancing EBP uptake among physical therapists. Little is known about the extent to which EBP is integrated in educational curricula in developing countries where the benefits of EBP are more acutely needed. This study sought to describe EBP education in Philippine physical therapy schools, including the challenges encountered by educators in teaching EBP.

**Methods:**

A national survey of higher education institutions offering an undergraduate degree program in physical therapy was conducted from August 2011 through January 2012. A 35-item questionnaire was developed to gather data on whether or not EBP was taught, specific EBP content covered and courses in which content was covered, teaching and evaluation methods, and challenges in teaching EBP. Data were analyzed descriptively.

**Results:**

The study had a response rate of 55.7% (34/61). Majority of the participating educational institutions (82%, 28/34) reported teaching EBP by incorporating EBP content in the professional courses. Among those that did not teach EBP, inadequate educator competence was the leading barrier. Courses commonly used to teach EBP were those on research (78.6%, 22/28), therapy planning (71.4%, 20/28), treatment skills (57.1-64.3%, 16-18/28), and undergraduate thesis (60.7%, 17/28). Various EBP contents were covered, with statistical concepts more frequently taught compared with critical EBP content. Lectures and journal reports were the usual teaching methods (96.4%, 27/28 and 89.3%, 25/28, respectively) while written examinations, completion of an undergraduate thesis, and oral reports (82.1%, 23/28, 78.6%, 22/28, and 78.6%, 22/28, respectively) were often used in evaluation. Students’ inadequate knowledge of statistics and lack of curricular structure for EBP were identified as leading challenges to teaching (75%, 21/28 and 50%, 14/28, respectively).

**Conclusions:**

Many physical therapy faculties across the Philippines are incorporating EBP content in teaching. However, there is arbitrary and fragmented coverage of EBP content and inadequate emphasis on clinically oriented teaching-learning and assessment methods. These findings suggest the need to design appropriate entry-level educational programs on EBP. Effective ‘educating the educators’ strategies are urgently needed and can have far-reaching positive repercussions on EBP uptake in physical therapist practice.

## Background

The call for a commitment to evidence based practice (EBP) in physical therapy (PT) has become more strident because providing evidence-based service to patients is considered a professional, moral and ethical obligation [1-3]. Use of EBP is believed to be an important means by which physical therapists can deliver safe and effective interventions, veer away from ineffective and potentially detrimental methods, and ultimately avoid wasting precious resources allocated to healthcare [4]. Given the strategic role of PT in ameliorating the worldwide burden of the disabling and lethal effects of non-communicable diseases [5], such a commitment becomes both urgent and important, and thus cannot be overemphasized. The World Confederation for Physical Therapy advocates that physical therapists implement EBP in patient care [3] and doing so requires competence in integrating best evidence from systematic research with one’s clinical expertise and patients’ values [6].

Surveys of physical therapists from both developed and developing parts of the world suggest a shared valuing of EBP among clinical practitioners [7-12]. Despite this, however, low implementation of EBP and high use of potentially biased evidence sources have been commonly reported [7,8,12,13]. A consistent barrier to routine use of EBP has been inadequate educational preparation [2,7,12] and consequently the need for effective education has been echoed widely across studies [7-12,14]. Continuing professional education programs have been demonstrated to impact positively on practitioners’ knowledge, but not necessarily on attitudes, skills, and engagement behaviors [15,16]. Individuals who receive entry-level education on EBP, however, have been shown to demonstrate positive EBP-relevant attitudes and self-efficacy [17] and a greater tendency to apply EBP clinically [8,12]. Cross-national survey evidence indicates that physical therapists utilize heavily their entry-level education in making practice decisions [18]. Therefore, preprofessional education may be the most opportune time to commence capacity building in EBP. This highlights the strong potential of effective early education as a tool to address the insufficient EBP uptake that pervades professional practice [2,19,20], especially in developing country contexts where such education can be of potent impact [19] and where the benefits of EBP are more acutely needed.

While in many developed countries education on EBP has been explicitly articulated in preprofessional curricula and models of curricular integration of EBP have been published, little is known about it in developing countries especially in the Southeast Asia (SEA) region where pervading practice and education contexts might be different. Education often mirrors the pervading practice environment and healthcare system, and policies that drive PT practice and healthcare impact PT education as well. For example, in the Philippines, clinical practice remains physician-directed under the law [21] and, expectedly, physical therapists have limited autonomy in decision making which may not galvanize efforts toward EBP upskilling. On close inspection of the core competencies and learning objectives in the required minimum curriculum for PT in the Philippines [22], there is an overall absence of a mandate to develop competence in EBP. This exposes an important issue on curricular relevance as entry-level programs and curricula in PT must be responsive to evolving practice patterns, scientific evidence, and a range of internal and external factors [23]. In spite of this, findings of a recent systematic survey reveal that Filipino physical therapists appear to experience some latitude in making practice decisions, are generally expected by their organization to apply research evidence in practice, and are reportedly receiving undergraduate education in research evidence uptake dimensions such as evidence searching, critical appraisal, and evidence integration [7]. This points to the value of EBP upskilling for these physical therapists as well as of exploring the extent to which students are being prepared to apply EBP. Although a local model of curricular integration of EBP has been reported recently in the literature [17], it represents the only PT program in the Philippines that is regulated by an independent charter and hence the model may be limited in depicting how EBP is covered across the remaining 88 HEI offering PT in the country.

Qualitative case study research has provided preliminary descriptions and insights on how EBP is taught in four educational institutions in the Philippines as well as challenges that educators face related to teaching EBP [E Gorgon, L Angeles, A Borras, K Collis, J Reyes, Unpublished research]. Given the lack of an educational substructure for EBP in the curriculum, the informants separately described similar themes of arbitrary integration of EBP content across the different preprofessional intervention and research courses, and fragmented use of teaching-learning and evaluation methods. The results of this qualitative research suggested the possibility that areas of overlap between research and EBP, and areas that were distinct to each one were not clear to the informants. This was underscored by a common theme of identifying the undergraduate thesis and thesis defense as key methods of teaching and evaluating EBP competencies respectively. These findings indicate educators’ insufficient education on EBP as a potential barrier that requires further investigation. As qualitative research is normally meant to allow for hypothesis generation and not hypothesis testing, quantitative research is warranted to determine the extent to which the findings are valid for a larger proportion of the population. This study sought to describe how EBP was incorporated and taught in entry-level PT educational programs in the Philippines as well as what challenges educators encountered in the process. Findings of this study can constitute crucial information that can be a basis for policies and interventions toward explicit and rationalized education of Filipino PT students on EBP. Lastly, the findings may be generalized to other SEA countries, with comparable practice and education contexts. Thus this study could be an important step in exploring ways of overcoming barriers related to inadequate physical therapist preparation in EBP.

## Methods

### Study design

This study employed a non-experimental descriptive survey design and was conducted from August 2011 through January 2012. The study was considered to be in accordance with principles of ethical research stipulated in the Declaration of Helsinki and approved by the institutional review board of the National Institutes of Health (project number NIH 2009-048).

### Study sampling

All higher education institutions (HEI) in the Philippines regulated by the government’s Commission on Higher Education (CHED) that had an active baccalaureate program in PT were included in the survey. At the time of the study, there were 88 such institutions of a total of 89 HEI that offered PT education in the country. One university, the Philippines’ national university, is governed by an independent charter and thus is not compelled to use the required curriculum for PT by the CHED. The longitudinally integrated EBP education program of this university which started in 2008 is described elsewhere [17]. Twenty-seven HEI were excluded following verification that there was either no student enrolment at any year level (n = 19) or student enrolment only in the first two year levels (n = 8). As prescribed in the CHED-regulated curriculum, the first two years of PT education comprise general education courses such as basic sciences, mathematics, arts and humanities, and social sciences, while PT foundational and professional courses (including supervised clinical practice) are mandated in the third through fifth years [22]. Finally, 61 HEI in the Philippines comprised the sampling frame. Institutional response was provided by either the school dean or department chairperson. Where either the dean or chairperson was unavailable to provide a response on behalf of the HEI, faculty duly selected by either the dean or chairperson accomplished the questionnaire.

In the absence of a definitive database of contact information (school or department heads’ names, mailing addresses, and telephone numbers) on the HEI, we pooled information obtained from the CHED and Professional Regulation Commission – Board of Physical and Occupational Therapy. This resulted initially in verified information for only 12 of the 88 HEI (13.6%). Information needed to communicate with the remaining HEI was gathered and verified through a variety of means that included locating school addresses and contact information in telephone company listings, Internet search engines and social networking sites; searching the database of the local association of rehabilitation science schools; searching participant databases of conventions and seminars organized by the national PT organization; personal site visits to schools situated in Metro Manila and nearby provinces; and seeking assistance from our professional and personal networks.

### Survey questionnaire

We developed a 35-item survey instrument (apply to the first author for a copy) based on multiple literature sources. The impetus for identifying teaching content, methods and challenges in EBP education was the 2009 qualitative case study research that made a preliminary exploration on how EBP was taught in undergraduate PT programs in the Philippines [E Gorgon, L Angeles, M Borras, K Colis, J Reyes, Unpublished research]. Items on which courses EBP was taught were lifted from the government-issued memorandum order that mandated the minimum content for PT curricula in the Philippines [22]. Items on specific EBP content were sourced from published work describing educational content and competencies critical to EBP in general [24] and evidence based PT in particular [4,23,25,26]. Items on teaching and evaluation methods, barriers to teaching EBP in the clinical practice courses, and challenges and facilitating strategies to teaching EBP were derived from the Philippines-based qualitative research by Gorgon et al. Items on challenges and facilitating strategies were augmented by literature on barriers to integrating EBP in the PT curriculum [23] and to EBP uptake among PT practitioners [7,8,12,14].

The original version of the questionnaire was pilot tested for relevance of content and clarity and ease of use on 6 rehabilitation therapists who had different levels of related experience in education and research. Revisions pertaining to clarity of certain terms and instructions, and questionnaire format and length were carried out on the basis of the specific comments from the pilot test participants. The resulting questionnaire comprised four sections that were utilized in gathering data on the: extent to which EBP was integrated in the curriculum, including the specific content and courses in which the content was covered (items 1-6); methods of teaching and evaluating student learning (items 7-8); challenges and facilitating strategies in teaching EBP (items 9-10); institutional and faculty characteristics, including access to continuing education and literature resources (11-35). The first section commenced with a question on whether or not EBP was taught in the curriculum and those with negative responses were asked to specify the barriers to teaching EBP. In addition, in asking what specific content was covered, the questionnaire asked participants to categorize the content they were not teaching as either undergraduate-appropriate or postgraduate-appropriate. Although most of the items were close-ended, the questionnaire included the option ‘Others, please specify’ where appropriate to avoid limiting the participants’ choices.

### Data collection

Survey questionnaires were distributed using a variety of methods such as electronic mail, facsimile, next-day courier service delivery, postal mail, and personal delivery to accessible HEI by the investigators and research assistant if required. As responses were meant to represent those of the institutions’, participants were explicitly instructed to consult the members of the PT faculty in replying to the questionnaire items. Weekly or fortnightly follow-ups were done based on participant preference. The participants were given multiple options for returning the questionnaire (electronic mail, facsimile, next-day courier service or postal mail to be reimbursed by the authors, and personal retrieval by a research assistant for accessible sites) to enhance the response rate. The questionnaire was accompanied by a cover letter that included salient information about the study and an explicit statement on the voluntary nature of participation. All participants were adequately informed of the purpose and value of the study, as well as of consent signified by returning the completed questionnaire. All data were de-identified and kept confidential. Printed questionnaires were stored in a locked drawer in the lead author’s office while electronically submitted questionnaires were kept in a password-protected electronic mailbox created specifically for the study.

### Data analysis

Data were entered in Microsoft Excel 2010 (Microsoft Corp, Redmond, WA, USA) and analyzed using an evaluation version of the Statistical Package for Social Sciences 15.0 (SPSS, Inc., Chicago, IL, USA). Where possible, participants were contacted either through telephone or electronic mail for clarifications regarding questionnaires that were returned with missing or conflicting data. Frequencies and percentages were determined for nominal-level data. Since data on ratio-level faculty characteristics were not normally distributed, medians and interquartile ranges (IQR) were reported. A research assistant encoded and de-identified all data, while two of the authors (EJRG and MDSB) provided independent counterchecking of the encoded data versus data on printed questionnaires for accuracy.

## Results

Of the 88 HEI in the Philippines with an undergraduate PT program regulated by the CHED, 61 (69.3%) met the inclusion criteria. Nineteen (21.6%; NCR = 4, Luzon Islands = 11, Visayas Islands = 2, Mindanao Islands = 2) were excluded for having an ‘active’ program but no student enrolment at any year level, while 8 (9.1%; Luzon Islands = 4, Visayas Islands = 1, Mindanao Islands = 3) were excluded because student enrolment was only either at the first or second year levels. Of the 61 HEI that were included, 34 returned the questionnaire for a 55.7% response rate. Sixteen (47.1%) were from the NCR, 10 (29.4%) from the Luzon Islands, 6 (17.6%) from the Visayas Islands, and 2 (5.9%) from the Mindanao Islands. The 27 non-respondents to the survey were 8 from the NCR, 12 from the Luzon Islands, 3 from the Visayas Islands, and 4 from the Mindanao Islands.

Majority of the participating HEI (82.4%, 28/34) reported teaching EBP in their PT program. These 28 HEI comprised 14 from the NCR, 7 from the Luzon Islands, 6 from the Visayas Islands, and 1 from the Mindanao Islands. The median number of PT faculty members in these HEI was 9 (IQR = 7). The median numbers of PT faculty members that taught research and EBP were 2 (IQR = 1) and 3 (IQR = 2), respectively. The characteristics PT faculties and HEI from which institutional responses had been extracted are detailed in Table 1. The 6 HEI (NCR = 2; Luzon Islands = 3; Mindanao Islands = 1) that did not teach EBP-specific content identified the following as the most common barriers: lack of teachers who had competencies to teach EBP (66.7%), lack of formal education on the EBP framework (66.7%), and lack of institutional mandate to include EBP in the curriculum (50%). Less commonly reported barriers were: inadequate curricular space for EBP (33.3%), lack of literature and technological resources to teach EBP (16.7%), inadequate knowledge of research methodology (16.7%), and inadequate understanding of statistics (16.7%). Lack of support from colleagues was not selected by any of these HEI as a barrier.

**Table 1:** Characteristics of physical therapy faculties and educational institutions teaching EBP (n = 28)

**Characteristic of members of faculties teaching EBP**	**n**^**a**^	**%**
Age range (yr)		
26 – 30	8	28.6
31 – 35	7	25.0
36 – 40	8	28.6
41 and higher	1	3.6
Gender distribution		
Males and females in equal numbers	7	25.0
More female than males	10	35.7
More males than females	10	35.7
Time since graduation (yr)		
1 – 5	8	28.6
6 – 10	11	39.3
11 – 15	6	21.4
Highest degree attained		
Bachelor	9	32.1
Physical therapy masters	3	10.7
Non physical therapy masters	12	42.9
Non physical therapy doctorate	3	11.1
Training on EBP as part of faculty academic preparation		
Yes, in undergraduate program	1	3.6
Yes, in graduate program	13	46.4
No	14	50.0
Attendance in continuing education on research		
Yes	22	78.6
No	6	21.4
Attendance in continuing education on EBP		
Yes	16	57.1
No	12	42.9
Average frequency of reading of literature in physical therapy/academic journals by faculty per week		
0	10	35.7
1	7	25.0
2	3	10.7
More than 2	2	7.2
Research teaching experience (yr)		
0	7	25.0
Less than 1	2	7.1
1 – 5	12	42.9
6 – 10	6	21.4
Physical therapy teaching experience (yr)		
Less than 1	1	3.6
1 – 5	15	53.6
6 – 10	11	39.3
Clinical practice experience (yr)		
Less than 1	6	21.4
1 – 5	15	53.6
6 – 10	5	17.9
Engagement in regular clinical practice		
Yes, less than 5 patients per week	10	35.7
Yes, less than 5 – 10 patients per week	4	14.3
No	14	50.0
Application of EBP in clinical practice		
Yes	7	25.0
No	21	75.0
Membership in professional practice organization(s)		
Yes	22	78.6
No	6	21.4
**Characteristic of educational institutions teaching EBP**		
Type of educational institution		
Public	3	10.7
Private	25	89.3
Location of educational institution		
Rural/Provincial	2	7.1
Urban/City	26	92.9
Average number of continuing education opportunities provided by educational institution per year		
0	2	7.1
1	2	7.1
2 – 3	18	64.3
More than 3	6	21.4
Access to physical therapy/academic journals		
Printed version	22	78.6
Online version	15	53.6
Access to Internet and online scientific databases		
At educational institution	16	57.1
At home/Other locations outside educational institution	14	50.0
Availability of resource person to assist in learning EBP at educational institution		
Yes	19	67.9
No	9	32.1

^a^Some frequencies not equal to 28 due to HEI non-response to some questionnaire items.

### Where EBP was taught in physical therapy curriculum

Content relevant to EBP was incorporated in the various foundation and professional courses in the third through fifth years of the standard government-mandated curriculum (100%), with two of these HEI indicating that a separate course on EBP was also offered. The specific courses that were most frequently utilized to cover EBP are listed in Table 2. The most commonly selected courses (at least 50%) were research methods and undergraduate thesis (Research 1 and Research 2, respectively), therapy planning courses (Seminar 1 and Seminar 2), and treatment skills courses (PT 4, Therapeutic Exercise 3, Therapeutic Exercise 2 and PT 2).

**Table 2:** Physical therapy courses where EBP was incorporated (n = 28)

**Undergraduate course**	**n**	**%**
Research 1: Introduction to Research and Research Proposal writing	22	78.6
Seminar 1: Clinical Correlations for Medical Conditions	20	71.4
Seminar 2: Clinical Correlations for Surgical, Neurologic, and Developmental Conditions	20	71.4
PT 4: Electrotherapy	18	64.3
Research 2: Research Implementation and Presentation	17	60.7
Therapeutic Exercise 3: Therapeutic Exercise for Surgical, Neurologic and Developmental Conditions	17	60.7
Therapeutic Exercise 2: Therapeutic Exercise for Medical Conditions	16	57.1
PT 2: Light, Thermal Agents and Hydrotherapy	16	57.1
PT 3: PT Examination and Evaluation	13	46.4
Therapeutic Exercise 1: Basic Therapeutic Exercise	12	42.9
PT 1: Introduction to PT and Patient Care	9	32.1
Orthotics and Prosthetics	7	25.0
Ethics in Physical Therapy	6	21.4
Organization and Administration in PT	4	14.3

One-half of the participants reported that their students had opportunities to apply EBP in supervised patient care during the final-year courses Internship 1 and Internship 2 while 14.3% did not know. Conversely, 35.7% said their students did not have such opportunities. Frequently identified by these HEI as hindrances to student learning on EBP during supervised clinical practice were lack of support for EBP uptake by the clinical placement sites (80%), lack of information resources in the placement sites (70%), lack of content in the placement sites’ clinical education program that focused on EBP (60%), and lack of interest in EBP among clinical supervisors (50%). Only 30% selected insufficient time as a barrier to teaching EBP in this context.

### What EBP content was taught

Majority of the participants (at least 50%) reported teaching 15 of the 23 specific EBP content listed in the questionnaire (Table 3). Least frequently taught content was in the area of critical appraisal of research evidence, in particular the use of instruments and understanding of certain methodological concepts (intention to treat analysis and power calculation, among others) to evaluate study validity, critical appraisal of specific study designs (systematic reviews and those on diagnosis and prognosis), and making sense of the evidence (evidence hierarchies and interpretation of forest plots, among others). Likewise, in the area of integrating evidence and communicating results, content on communicating results of evidence appraisal to patients at an appropriate level of language was taught in less than 50% of the participating HEI. However, except for the specific content on types of clinical questions and critical appraisal of studies on intervention, diagnosis and prognosis, most of such content were considered by the HEI to be needed in and suitable for undergraduate-level PT education.

**Table 3:** Specific EBP content taught in the physical therapy curriculum (n = 28)

**EBP content**	**n**	**(%)**
Asking clinical question and searching for evidence		
Formulating clinically relevant questions using PICO format^a^	16	57.1
Different types of clinical questions to guide literature search^b^	20	71.4
Constructing focused search strategy^a^	19	67.9
Locating clinical evidence using electronic databases^a^	19	67.9
Critically appraising evidence		
Commonly used study designs and their major strength and limitations^a^	21	75.0
Assessing relevance of study design to clinical question^a^	16	57.1
Hierarchy or levels of evidence^a^	13	46.4
Differences among narrative review, systematic review and meta-analysis^a^	16	57.1
Difference between clinical and statistical significance^a^	15	53.6
Interpreting results of statistical procedures such as t tests, chi-square tests^a^	20	71.4
Interpreting results of statistics such as p-value, confidence interval^a^	17	60.7
Understanding sensitivity and specificity, number needed to treat, odds ratio^a^	16	57.1
Understanding intention to treat analysis, power calculation^a^	10	35.7
Use of appraisal tools to assess validity^a^	12	42.9
Ways in which study validity can be threatened^a^	16	57.1
Difference between internal and external validity^a^	19	67.9
Critical appraisal of systematic reviews^a^	9	32.1
Interpreting forest plots in systematic reviews^a^	5	17.9
Critical appraisal of studies about intervention^b^	14	50.0
Critical appraisal of studies about diagnosis^b^	12	42.9
Critical appraisal of studies about prognosis^b^	12	42.9
Integrating evidence and communicating results		
Deciding on appropriate clinical decision considering patient’s needs and treatment preferences^a^	18	64.3
Communicating results of appraisal at level appropriate to individual patient^a^	13	46.4

^a^Considered appropriate for undergraduate-level education by majority (at least 50%) of participants who did not teach this content in undergraduate curriculum.

^b^Considered appropriate for postgraduate-level education by majority (at least 50%) of participants who did not teach this content in undergraduate curriculum.

### What methods of teaching and evaluation were employed

Table 4 details the teaching and evaluation methods that were most frequently employed in educating students on EBP. The most highly utilized methods for teaching EBP content were the lecture (96.4%) and journal report (89.3%), while the use of workshops or seminars on critical appraisal instruments (17.9%) and electronic evidence searching (10.7%) were least applied. More than half identified thesis advising (64.3%) and thesis presentation or ‘defense’ in a research colloquium (57.1%) as teaching methods of choice. Evaluation of student learning of EBP content was most frequently accomplished through written examinations, completion of an undergraduate thesis, oral reports, and written assignments. It was noted that 78.6% made use of the undergraduate thesis as a means of assessing EBP competence.

**Table 4:** Methods of teaching-learning and evaluation employed (n = 28)

	**n**	**(%)**
**Teaching method**		
Lecture	27	96.4
Journal report	25	89.3
Mentoring during thesis advising	18	64.3
Small group discussion	18	64.3
Guided literature search	16	57.1
Research colloquium	16	57.1
Critical appraisal of scientific articles	14	50.0
Workshop/Seminar on critical appraisal instruments	5	17.9
Workshop/Seminar on electronic searching	3	10.7
**Evaluation method**		
Written examination	23	82.1
Completion of undergraduate thesis	22	78.6
Oral report	22	78.6
Written assignment	21	75.0
Oral examination	11	39.3
Class recitation	11	39.3
Poster presentation	6	21.4

### What challenges were encountered and what strategies facilitated teaching

Of the different challenges that the educational institutions faced in teaching EBP (Table 5), inadequate knowledge of statistics in students (75%) and lack of curricular structure for teaching EBP (50%) were the most reported. Other challenges were related to a lack of structure for evaluating competencies in EBP, lack of student interest in EBP, inadequate preprofessional education of the faculty on EBP in general and inadequate critical appraisal skills and ability to configure postgraduate education into EBP education for undergraduate students in particular, and inadequate continuity of learning on EBP into the final year when students underwent clinical placements. The participants identified strategies that they had used to be able to teach EBP in the curriculum (Table 5). The most common strategies were to incorporate research evidence in lectures delivered in the various courses (78.6%) together with modification of course syllabi toward including EBP content (64.3%) and provision of hypothetical clinical cases that students could use in discussing EBP (64.3%). Protected time for faculty to engage in research as a means of upskilling in EBP was least frequently adopted by the HEI (21.4%).

**Table 5:** Challenges and strategies in teaching EBP (n = 28)

	**n**	**(%)**
**Challenges to teaching EBP**		
Students’ inadequate knowledge of statistics	21	75.0
Lack of curricular structure for teaching EBP	14	50.0
Lack of curricular structure for evaluating EBP competencies	13	46.4
Faculty’s inadequate EBP competence gained in undergraduate education	13	46.4
Students’ lack of interest in EBP	12	42.9
Faculty’s difficulty in using EBP knowledge gained in postgraduate education in teaching undergraduate students	10	35.7
Faculty’s limited ability to critically appraise literature	10	35.7
Inadequate learning activities in supervised clinical practice courses to allow continuity of learning	10	35.7
Lack of professional journals in library	9	32.1
Faculty’s lack of confidence in teaching EBP	8	28.6
Faculty’s inadequate skills in searching Internet databases	6	21.4
Faculty’s insufficient computing skills	5	17.9
Lack of institutional mandate to include EBP in curriculum	5	17.9
Lack of access to Internet databases	5	17.9
Lack of time/space in curriculum to incorporate EBP	4	14.3
Lack of sufficient evidence to answer clinical questions	4	14.3
Lack of Internet access at educational institution	3	10.7
Faculty’s negative attitude toward teaching EBP	2	7.1
**Strategies to facilitate teaching of EBP**		
Incorporating research evidence in lecture	22	78.6
Modifying course syllabus to incorporate EBP	18	64.3
Providing hypothetical clinical cases to students to facilitate EBP discussion	18	64.3
Providing journal articles that students can use	13	46.4
Strengthening student knowledge of principles of statistics	11	39.3
Encouraging students to incorporate research evidence in oral reports	7	25.0
Mentoring junior faculty members on EBP	7	25.0
Providing faculty protected time to engage in research	6	21.4

## Discussion

This is the first systematic survey to report on EBP education in PT educational institutions in a developing country focusing on the SEA region. This study provides evidence that faculties in the Philippines have been including EBP, albeit arbitrarily and variably, in the educational preparation of physical therapists. Agarwal et al. [19] opined that effective education might be the most powerful strategy toward ameliorating EBP barriers in an Asian context. Appropriately structured educational programs and adequately equipped education providers are imperative if effective education in EBP is envisaged. The study thus adds to the knowledge by describing EBP education and challenges in teaching EBP in a SEA country. The findings justify the need for timely curricular enhancements and tailored interventions toward ‘educating the educators’.

The EBP-teaching HEI seemed to have integrated EBP in the PT curricula in varying degrees and ways. This might be expected given the lack of a policy mandate that requires HEI to develop essential knowledge and skills in EBP. It may be indicative of the educators’ positive attitudes toward EBP and increasing sense that EBP is not optional but rather expected of physical therapists on entry to professional practice. Slavin [26] has elucidated that the recent emphasis on EBP has challenged PT educators to learn the evidence based medicine principles, transform the principles for use in PT practice, and embed EBP in curricular content. Moreover, EBP concepts and summaries of contemporary research have become pervasive in obligatory reference textbooks in local educational institutions, such as those by O’Sullivan and Schmitz [27] and Kisner and Colby [28].

Teaching EBP has been done through establishing an EBP focus throughout the curriculum, incorporating it in a research course, or threading it in clinical management courses [20]. Most of the HEI integrated EBP in the research courses (including the research implementation and presentation course), clinical decision making courses (the ‘clinical correlations’ courses), and treatment courses (specifically the courses on electrophysical agents and therapeutic exercises). Although incorporating EBP in research courses is common, the underpinning rationale that, to be able to use EBP, one must first have the ability to design, complete, and disseminate a research project has been greatly challenged [1]. Indeed, although specific research knowledge and skills form a critical foundation for EBP [4,24-26], one can engage in EBP without necessarily having the ability to generate an original research first. The less frequent integration of EBP in the assessment (13 of 28 HEI) and ethics (6 of 28 HEI) courses is noticeable. Teaching EBP in the assessment course would be a highly opportune time to guide students through selecting and interpreting evidence-based tests and measures. Incorporating EBP in the ethics course can promote a substantial appreciation in students of the relationship between EBP and ethical practice. The low frequency of teaching evidence-based assessment mirrors the weak emphasis on assessment as a physical therapist responsibility area among practitioners [E Rotor, Unpublished research] and may explain the very infrequent use of standardized outcome measures among Filipino physical therapists [E Gorgon, A Lugue, M Magsino, Unpublished research].

Although there was a clear attempt to teach various relevant EBP content, coverage of content appeared to be generally uneven and partly incongruous with known essential EBP knowledge and skills [4,24-26]. For example, the greater emphasis on interpretation of statistics compared with formulating clinically relevant questions, assessing the relevance of study designs, critical appraisal of evidence, and communication of evidence to patients suggests that critical elements in EBP as a decision making framework are being missed. This is consistent with the participants’ identification of students’ lack of knowledge in statistics as the principal barrier in teaching EBP. This might result in inadequate ability to apply EBP as a decision making framework once the students become independent clinical practitioners.

When asked to classify the specific content that they were not teaching as either undergraduate-appropriate or postgraduate-appropriate, most of the participants deemed almost all content as undergraduate-appropriate. In a two-round Delphi process involving experts from developed and developing countries, Yousefi-Nooraie et al. [24] established that statistics and critical appraisal of studies other than those on interventions would be appropriately covered in advanced-level EBP. This implies an inability to distinguish relative complexity levels of specific EBP content among the participants. It may also indicate a perception among the participants that EBP is largely dependent on technical knowledge in research. Such a perception can obscure the other critical EBP dimensions of clinical expertise and patient values and preferences. In all, these findings underscore the need for a deeper understanding of EBP among Filipino PT educators. We disagree with Yousefi-Nooraie et al.’s finding that communicating evidence to patients would be best taught at an advanced level. Communicating evidence to patients is an essential skill [26] that can allow physical therapists to infuse their patients’ ‘voice’ into clinical decision making. Thus learning this skill early at the undergraduate level can help practitioners avoid a piecemeal approach to implementing EBP. Moreover, the non-availability of regular structured continuing education programs on EBP in the Philippines and low feasibility of enrolling in a postgraduate masters program for most Filipino practitioners strongly support the teaching of this essential skill at the undergraduate level.

The following comment volunteered by one school dean was telling in terms of what ‘teaching EBP’ probably meant to the faculties: ‘Although I indicated that we do evidenced (sic) based practice here in our setting, much of the data being taught are those that have been published in reputable textbooks that have extensive literature review and had evaluated literature based on EBP guidelines. We also use Clinical Practice Guidelines with the presumption that the EBP process have (sic) also been applied in its (sic) formulation’. While the attempt to incorporate content culled from pre-appraised literature such as clinical practice guidelines is encouraging, it does not necessarily translate into students’ skills in locating evidence and communicating and integrating evidence. Some authors have supported the development of a limited set of skills in acquiring and applying pre-appraised evidence as ‘evidence users’ [29,30] to increase the likelihood of evidence uptake in routine clinical practice. Gorgon et al. [7] allude to this as a viable option given the PT practice context in the Philippines, especially if the goal is to promote widespread evidence uptake. However, of the 28 HEI that reported teaching EBP, only 3 (10.7%) provided their students workshops on evidence searching. Locating and accessing credible evidence, making judgments about the soundness of the evidence, and finding resources for EBP upskilling, are critical evidence consumption skills [25,26,31]. These skills must be emphasized if at least ‘evidence user’ competence is to be a realistic learning outcome in students. This further raises the point of why it is important to define minimum EBP competencies and provide a realistic framework for developing EBP competence in the prescribed curriculum for PT in the Philippines.

The participating HEI’s over-reliance on lectures, reporting journal article summaries, and other teaching methods will not likely develop the higher-order thinking skills necessary for evidence based decision making. This might either result in the development of a superficial appreciation of EBP or create misconceptions on what it is and how the process is carried out. Health practitioners’ level of confidence in simply knowing EBP methodology may not necessarily translate into ‘real world’ implementation of EBP [32]. Intervention studies have demonstrated that EBP education may not be optimally attained through short courses or standalone and ‘one-off’ workshops because, while knowledge often improves [15,33], participants are not likely to change their attitudes, skills, and usual practice [15,16]. Since EBP involves complex knowledge and skills that take time to develop, it follows that EBP education should integrate multiple factors, comprise multiple layers of teaching and evaluation, and allow students time to develop their thinking and decision making skills. A recent model of undergraduate EBP education for PT advocates the value of faculty-student collaboration in tackling real patient cases and has reported positive preliminary outcomes [17]. The model highlights both the insufficiency of conventional methods of instruction [1,26,34] as well as the value of role modelling by faculty in EBP education [34,35]. There is a need to discover how students can be educated effectively and creatively on translating what they learn in the classroom into sound clinical decisions.

The use of thesis writing and thesis defense as two of the most frequent methods of evaluating EBP competence is revealing in that it represents a possible inadequacy in awareness of both common and distinctive features of research and EBP [E Gorgon, L Angeles, M Borras, K Colis, J Reyes, Unpublished research]. This assertion is strengthened by the finding that two of the usual methods for teaching EBP were thesis advising and research colloquia or presentations. There may be a pervasive perception that, since EBP requires good foundational knowledge in research, then EBP competence and research competence are the same. Such perception might be deeply rooted in the lack of EBP competence gained in undergraduate education and insufficient continuing education on EBP reported by approximately half of the participating faculties. This clearly proffers the need to clarify both the relatedness and distinctiveness of the knowledge and skills sets for research consumption in EBP, and knowledge generation in research. Further, it highlights the need to deepen EBP-relevant knowledge and skills among Filipino educators.

With only 50% of the 28 HEI reporting that their students had opportunities to learn EBP during supervised clinical practice, it was possible that the clinicians who mentored students at clinical placement sites did not utilize EBP or that EBP might have been viewed as a mere academic exercise. The barriers identified by most of these HEI mirror the barriers to uptake of EBP in clinical practice. Low research evidence uptake in Filipino physical therapists has been described in a recent survey [7]. This has important repercussions because EBP cannot be solely an academic pursuit and may be learned effectively if applied in clinical case scenarios or in students’ clinical practice [15,17,20,26,34,35]. Thus, supervised clinical practice in the students’ final year might be a most opportune time to teach students how to integrate best-available research evidence and clinical experience. Students have been shown to rely heavily on supervisors’ opinion in answering clinical questions [36]. Therefore, if their supervisors are not EBP-competent or do not serve as role models of evidence-based decision making, then this can have a deleterious effect on student learning [35]. To elevate the current level of EBP education of Filipino PT students, EBP should be integrated in the clinical education infrastructure and clinical educators must be upskilled on EBP. This also points to the need for better cooperation between HEI and clinical placement sites for concerted identification of facilitators and barriers, and implementation of synergistic educational strategies [26].

The most potent impetus that can drive widespread and substantial integration of EBP in the Philippine PT curricula would be an explicit articulation of specific learning objectives and content on EBP in the minimum standards for education enforced by the CHED. The lack of an educational infrastructure for EBP in the prescribed curriculum was the second most identified challenge by the EBP-teaching HEI. However, the process of policy revision takes time. For example, the CHED revision of the 1998 prescribed curriculum was completed and disseminated to take effect in 2006 [22]. There is an urgent need therefore to help PT educational institutions in threading and defining EBP in their existing curricula. Examples in the literature suggest that this can be feasible and can have a positive impact on students’ attitudes toward the value of EBP in ‘real world’ practice and self-efficacy in key knowledge and skill areas in EBP. Ross and Anderson [1] reported their integration of EBP in existing research methods and clinical decision making courses. Portney [34] described a clinically integrated learning program using a variety of teaching-learning and evaluation strategies to highlight the inextricable link between research and practice. Rotor and Gorgon [17] amalgamated elements from different EBP education models to expand the research methods course and longitudinally thread EBP from academic courses through supervised clinical practice courses. This strategy was meant to expose students to the intricacies of ‘real world’ decision making that is central to EBP. The way forward would be for further research to investigate viable ways of integrating EBP in the PT curriculum which inevitably must be grounded on contextual realities of the HEI.

Findings of this study have important ramifications on faculty development in Philippine PT schools. Academic and clinical faculties’ EBP competence must be continually upgraded because they have the critical role of priming future health professionals to be responsive to changes in practice, the evolving state of the science underpinning the profession, and the needs of society. Many of the participating PT faculties in this study, however, were inadequately prepared to teach EBP and not many had ongoing clinical practice that could be optimized for upskilling in EBP. For example, among those that taught EBP 50% had no educational preparation on EBP, 43% had not attended continuing education on EBP, and only 18% read scientific literature more than once per week. Although all had clinical experience to some extent, only 50% were engaged in regular (weekly) clinical practice and 25% had applied EBP in patient care. Among those that reported not teaching EBP, lack of educational preparation on EBP and inadequate competence in teaching EBP were the most frequently selected barriers. These clearly indicate the need to investigate the educational needs of PT faculties in EBP and teaching EBP. As well, the available within-HEI infrastructures and resources that can support EBP need to be explored toward creating effective tailored ‘educating the educators’ interventions.

### Limitations

The survey questionnaire was not formally tested for validity and reliability, although it was grounded on relevant literature and underwent pilot testing to enhance its utility. As with any cross-sectional survey design, the ability to make a deeper exploration of issues that underpin the findings may be limited. For example, we were unable to probe into how the process of making clinical decisions based on patient needs and preferences was taught. Approximately two-thirds of the HEI that taught EBP reported covering this content and one-half believed their students experienced opportunities to use EBP in supervised clinical practice, but the data could not allow for sufficient inferences regarding the real extent and nature of such coverage. Individualized decision making is a complex process that involves careful consideration of multiple contextual factors and therefore requires multifaceted teaching-learning methods to develop skills in students. Given the strong reliance of the faculties on teaching-learning methods that typically target lower-order thinking skills, we hypothesize that the requisite complex skills for evidence based decision making are not being realized currently in learners. An important next step would be to clarify how Filipino PT students are educated on the process of using evidence in decision making. Moreover, how such education carries over to and impacts their decision making as clinical students initially and as clinical practitioners later on requires attention.

Although the return rate was modest at 55.7%, generalizations from the study could be aided by the participation of educational institutions from all four major geographical regions in the Philippines. The strong encouragement given to all participating HEI to consult their respective faculties in replying to the questionnaire items increases our confidence that the responses closely reflect the true institutional responses. It is possible that the results may represent an overestimation of the extent to which EBP was taught because those that returned the questionnaire might be the HEI that valued EBP enough to teach it to students. Conversely, those that did not return the questionnaire might have been the HEI that did not support EBP sufficiently to teach it in the curriculum. In the absence of accessible and reliable data on the non-responding HEI, it would be difficult to make inferences regarding characteristics that might differentiate HEI that taught EBP from those that did not. ‘Self-reporting’ by the HEI might have also inflated the reported extent of coverage of EBP since it has been suggested to provide a more positive picture of a phenomenon compared to the actual status [14,32].

## Conclusions

Although the development of EBP-specific competence is not currently required in physical therapist education in the Philippines, many educational institutions report teaching EBP-relevant content. Teaching of EBP, however, has been arbitrary and variable overall, and at best fragmented in content and method of delivery and evaluation. The findings are highly suggestive of the need for explicit articulation and longitudinal threading of EBP in learning objectives in the required minimum curriculum for PT. Also, faculties especially those who supervise students in their clinical practice must be adequately upskilled in applying EBP and teaching EBP to allow them to potentiate their ability to educate students on EBP. The next important steps are to assess educators’ learning and teaching needs in EBP toward designing relevant ‘educating the educators’ programs and explore ways of building a suitable infrastructure for EBP education in Philippine HEI. The apparent valuing of EBP among PT faculties augurs well for future policies and interventions that would ameliorate key barriers to physical therapist preparation on being effective evidence consumers in ‘real world’ practice.

## Abbreviations

CHED: Commission on Higher Education; EBP: Evidence based practice; HEI: Higher education institution; IQR: Interquartile range; NCR: National Capital Region; PT: Physical therapy; SEA: South East Asia.

## Competing interests

The authors declare that they have no competing interests.

## Authors’ contributions

EJRG, MDSB, and ATM conceived and designed the study. All authors contributed in administering and retrieving the questionnaires. EJRG and MDSB analyzed and interpreted the data. EJRG prepared the manuscript with specific contributions from MDSB and ATM. All authors contributed in revising prefinal versions of the manuscript and approved the final version of the manuscript. MDSB and ATM contributed to the project when they were instructors at the Department of Physical Therapy, College of Allied Medical Professions, University of the Philippines Manila.

## Pre-publication history

The pre-publication history for this paper can be accessed here:

http://www.biomedcentral.com/1472-6920/13/154/prepub
